# Education for patients with limb loss or absence: Aging, overuse concerns, and patient treatment knowledge gaps

**DOI:** 10.3389/fpsyg.2022.953113

**Published:** 2022-09-27

**Authors:** Dawn Finnie, Joan M. Griffin, Cassie C. Kennedy, Karen Schaepe, Kasey Boehmer, Ian Hargraves, Hatem Amer, Sheila Jowsey-Gregoire

**Affiliations:** ^1^Kern Center for the Science of Health Care Delivery, Mayo Clinic, Rochester, MN, United States; ^2^Transplant Center, Mayo Clinic, Rochester, MN, United States; ^3^Knowledge and Evaluation Research Unit, Mayo Clinic, Rochester, MN, United States; ^4^Knowledge Encounter Research Unit, Mayo Clinic, Rochester, MN, United States

**Keywords:** vascularized composite allotransplantation, education, qualitative interviews, standardized tools for evaluation, VCA

## Abstract

The goals of vascular composite allotransplantation (VCA) for hand are to maximize functional status and psychosocial wellbeing and to improve quality of life. Candidates are carefully vetted by transplant programs through an extensive evaluation process to exclude those patients with contraindications and to select those that are most likely to attain functional or quality of life benefit from transplant. Patient choice for any treatment, however, requires that candidates be able to understand the risks, benefits, and alternatives before choosing to proceed. This study aimed to understand patients’ knowledge and perceptions about treatment options for hand loss, including hand transplant. This study will be used to inform a standardized education approach and develop conversation aids for use by clinicians and patients throughout the treatment decision process. Ten individuals who had experienced hand amputation or had congenital limb loss were interviewed to better understand previous and current decisions about treatment, experiences in adjusting to their treatment, and perceptions about hand VCA. From this qualitative interview data, four findings emerged: (1) knowledge and education around VCA as a treatment option; (2) adaptation of individuals with limb loss; (3) fear of risk associated with transplantation; (4) issues of aging and overuse injuries to existing limbs. Results suggests that there is opportunity for expanding education about all treatment options for patients with new loss, long-term loss, and congenital limb loss. Establishing a baseline of knowledge about all options–prosthetics, rehabilitative strategies, and VCA—can help patients evaluate their values and goals of treatment. Issues associated with aging, including overuse and injury, and adaptability over the life course should be included in considerations about treatment choices. Data indicate the need for routinely assessing patient preferences about treatment choice so patients can plan for their future as they adapt and age and as technology for treatments change. To assure that thorough information is provided for current and future decision-making, education about treatment choices and selection procedures for VCA should be standardized.

## Introduction

In 2005, the worldwide prevalence of limb amputations was 1.6 million, and projections suggest that number may double by 2050 (Ziegler-Graham et al., 2008). Treatment options for upper extremity amputations include prosthesis, replantation, and vascular composite allotransplantation (VCA), with prothesis the most common treatment option. VCA is still rare but is becoming a feasible surgical alternative to replantation and prosthesis for some amputees.

The first hand transplant was performed in 1998 (Dubernard et al., 1999). Since then, more than 113 VCA hand procedures have been performed worldwide on 76 patients, some unilateral and some bilateral (Alolabi et al., 2017). Primary goals of hand transplantation are to maximize functional status, emotional status, and quality of life. Patient selection for hand transplant is complex and requires a thorough evaluation to assess the impact, risks, and benefits for each individual and to understand if hand transplantation will improve their quality of life. Developing standardized tools for identifying, evaluating, and assessing potential candidates and standardized approaches for patient education are key to assuring that selected candidates will benefit from transplant (Kumnig et al., 2014; Jowsey-Gregoire and Kumnig, 2016).

As an alternative to replantation and prosthesis, VCA can offer the potential for better sensation outcomes than prosthetics (Pasquina et al., 2006; Schuind et al., 2007). Data from all hand transplant recipients suggests improved protective sensibility of the recipients assessed, with 91% having tactile sensibility, and 82% regaining partial two-point discrimination sensibility. This improved function enabled independent living and, for some, return to full-time occupation (Shores et al., 2017). However, in a study comparing functional and psychological outcomes of hand transplant versus prosthetic users, no significant differences were found in functional outcomes between the two, but transplant patients did have higher scores in psychological areas of “role-physical,” “vitality,” “role-emotional,” and “mental health” (Salminger et al., 2016).

While the option of VCA may provide functional, psychological, social, and even cosmetic benefits for some, there are significant risks associated with lifelong immunosuppression (Dubernard et al., 1999; Hautz et al., 2020). Challenges also exist for VCA candidates around financial burden, missed work, and insurance coverage (Chung et al., 2010; Alolabi et al., 2015, 2017). An economic analysis of hand transplants indicated hand transplant to be significantly more costly than prosthesis and concluded that a unilateral hand transplant would not be considered cost-effective (Chung et al., 2010; MacKay et al., 2014). The decision-making process for treatment options for people with amputation is a balance between weighing risks, burdens, and benefits for all treatments. Seeking to improve functionality is a universal goal across treatment choices, but the broad aspects of quality of life are less frequently assessed. Hand transplant may improve quality of life, but VCA also includes a complex set of life-long risks. Understanding the broad spectrum of quality of life issues is critical for being able to weigh the risks and benefits. Patient satisfaction, for example, is one aspect of quality of life that is significantly related to function (Hautz et al., 2020), but other aspects, such as psychosocial function, wellbeing, and meaning making, are less commonly assessed. Bahler (2019) has commented on factors essential to quality of life that are currently not included in most patient assessments: “…our capacity to make meaning, particularly in relation to temporality, embodiment, and intersubjectivity. Without attention to these features of the human condition, assessment of hand transplant recipients’ functional capacity and psychological sense of satisfaction will remain incomplete”(Bahler, 2019).

With the expected increase in prevalence of limb loss and the complexity of risks and benefits for treatment options, data are needed to inform the development of standardized tools to assess a broad set of quality of life factors and develop patient education materials that can promote shared decision making. In this study, we aim to fill this gap by utilizing qualitative research methods to better understand the values, attitudes, and expectations of treatment for people with limb loss that may be associated with future quality of life and address patient education needs.

## Materials and methods

### Study design

To understand factors integral to treatment decision-making and education for potential VCA-eligible individuals (age range = 38–72), we conducted semi-structured interviews between March 2019 and March 2021 with ten individuals with upper extremity loss who were potentially eligible for upper limb transplantation. Interviews were conducted with participants from a variety of backgrounds, and great variation in their path to extremity loss, including congenital loss, active-duty military loss, and loss due to hospital-acquired sepsis.

### Source of participants

Purposeful sampling was initially used to identify participants through medical record review of patients with limb absence. Snowball sampling was used for further recruitment as was outreach to amputee advocacy organizations. Participants were contacted *via* recruitment letter or email by the study coordinator to explain the study purpose and request participation. If no response to the initial contact was received, a follow-up contact was made. A study coordinator contacted interested participants to schedule interviews with one of three interviewers (KS, JG, and DF). Interviewers included two experienced Ph.D. trained qualitative researchers and one experienced Master’s degree trained qualitative researcher. Recruitment and data collection concluded when thematic saturation was reached.

### Source of data

The semi-structured interview guide was created to capture the lived experience of individuals with limb absence, quality of life, and how they approach healthcare decisions. It included questions on how they came to have an upper limb extremity loss, discussed their life without an upper limb(s), their experience or knowledge of prosthetics, and thoughts and interest around hand transplantation. The guide was iteratively adjusted to fit each individual’s unique situation and to gather the most detailed and pertinent data from each participant. For example, questions were modified to capture the lived experience of an individual with congenital limb loss and modified again for a person with a military injury resulting in limb loss, as the treatment options and decision-making process may also be different.

All interviews were digitally recorded, transcribed verbatim, and de-identified (removing names and other identifiable features). Recorded interviews were stored on a secure server for analysis in Nvivo (Qi Li Group Pty Ltd, 2020).

### Data analytic approach

The qualitative research team (DF and JG) reviewed each transcript as they became available and listened to the interview audios. They independently read the interviews multiple times and listened to the audios to become immersed in the data. Initial key concepts were captured from the data and supported by the interpretive qualitative framework of medical anthropology (Lambert and McKevitt, 2002; Bernard, 2011), emphasizing the lived experience of individuals through the narrative they share about their healthcare journey. Interviews were semi-structured but allowed for flexibility to probe new or emerging issues.

Descriptive coding of interview transcripts ran concurrently with data collection, consistent with iterative thematic analysis (Corbin and Strauss, 2015). After each new interview, DF, a qualitative research analyst, and JG, a health services researcher, with qualitative and mixed methods expertise, independently read and analyzed each transcript and met regularly to reflect on the data and discuss the concepts, themes, and codes. With the review of each transcript and constant comparison across transcripts, a deeper understanding of the themes was reached. Concurrent analysis with data collection allowed for iterative interaction between data and analysis in order to enhance reliability (Morse et al., 2002). Codes and themes were then organized and analyzed through interpretive thematic analysis (Corbin and Strauss, 2008), and each transcript was entered into Nvivo. Data were organized by code and theme.

We concluded data collection after ten interviews because no new themes emerged.

## Results

As shown in Table 1, three women and seven men, ranging in ages from 38 to 72 were interviewed. Participants were individuals with varying amounts of time since initial extremity loss, from 1 to 64 years. Five had unilateral limb loss and five had bilateral limb loss. None were VCA recipients.

**TABLE 1:** Hand absence interview demographics.

	Gender	Age (years)	Years with amputation	Cause of extremity loss
1	F	64	4	Sepsis/bilateral.
2	M	50	22	Work injury/unilateral.
3	M	64	64	Congenital/partial bilateral.
4	M	38	2	Infection/bilateral.
5	M	65	40	Farm accident.
6	M	71	15	Arm shot off in active duty and reattached.
7	M	49	49 (additional wrist amputation 2 years)	Born without hand but had a bit of wrist. Two years ago, had squamous cell cancer and removed wrist.
8	F	72	1	Sepsis–bilateral. Lost all toes.
9	F	67	64	Gunshot accident as a child. Arm amputated about 1” below elbow.
10	M	59	1	Skin cancer. Amputation of both arms. Skin cancer removed from ear, face, back.

As common concepts and ideas emerged through the interview and coding process, four themes were identified: (1) awareness and knowledge of transplantation; (2) aging with amputation and/or VCA and overuse and injuries of existing limbs; (3) fear of risk associated with transplantation; and (4) contentment and adaptability to limb absence in long-term amputees. Themes included factors impacting knowledge about VCA and treatment decision making. Each subsequent interview helped to explore more depth into initial themes.

### Adaptability to limb absence

Participants living successfully with upper extremity loss/absence identified ways they have adapted their jobs, hobbies, and lives, and continue to adapt as they age.

These individuals have been living successfully with various forms of prosthetics and have adapted their routines. Participants living for longer than 15 years (*n* = 6) with an upper extremity absence also noted that they had adapted their lives to incorporate their limb loss. They had completed education and degrees, changed careers and started businesses, incorporated adaptations to their hobbies and outside activities, maintained family and social relationships, and spoke about their resilience and adaptability as assets. Interestingly, none of the participants interviewed referred to themselves as disabled or handicapped, and some rejected the terms when used in reference to themselves.

“…*you know, if you’ve maybe got just a couple of fingers, there have been many times I wish I had just one finger on the end of my arm, just one, to be able to do something. That’s a frustration of mine. They’re saying, ‘Well don’t you want the whole hand?’ Not necessarily. The way I do things now, a lot of times, all I need is that one damn finger.” (male, 64, congenital loss of one arm).*

*“Yeah. I’ve got stuff. I mean we go kayaking. I got stuff to hold the paddle. I’ve got stuff for the ATV. I got some things that hold guns when I shoot that will hold the forearm. Yeah cause that’s what I do at work. So once in a while, I’ll make something that I can’t buy somewhere, and I’ll just make my own, you know. Like even for like when we go deer - for cleaning deer, I got one with a knife on the end I can hold and skin it, you know, and stuff - just weird, different - I got one of the big vice grips so I can clamp things and heat ‘em with a torch, just different things.” (male, 65, lost hand 40 years prior in farming accident).*

*“I learned how to put screws in my cutting board, put the chicken on it, and that holds it. I have attachments to open bottles with if I need to. I have a one-handed syringe with the CO2 to open a wine bottle, everything adaptive.” (male, 71, arm shot off in active duty 15 years prior and reattached).*

*“I do mechanic work my whole life. I was born without my hand originally. So, I guess I’ve learned to adapt very well. I did have a prosthetic when I was younger and grew up through the Shriners. But then, as I got older and started to do more, I found–I was very fortunate I had the left arm all the way down, including the wrist. So, milking cows, driving equipment and stuff, I found that I could do better without the prosthetic just because I had the skin, I had the length, I could reach and do everything. I could balance the milkers better. I could drive the equipment better without the big rigid hook being in the way. I know technology has changed, obviously, quite a bit in 30 years [laughs].” (male, 49, congenital hand, additional wrist amputation 2 years prior due to cancer).*

*“I’ve gotten so used to living without my arm, my prosthesis almost gets in the way. It almost gets in the way because I’ve learned how to do so much in my armpit and holding my stump down and all kinds of stuff*… *I’ve learned how to do stuff so much without my arm, now my arm is like–I’m learning how to re-use my arm and keep it out of the way. When I need it, I need it. I haven’t needed it for nothing yet.” (male, 59, skin cancer led to arm amputation one year prior).*

### Lack of awareness or knowledge of vascular composite allotransplantation as an option

Participants’ knowledge about upper limb transplantation was varied. Some were aware of cases of upper extremity transplantation but had little knowledge about details of eligibility. Some had an understanding the risk and benefits but did not consider it an option for themselves due to age or the burden of immunosuppression. Some had no knowledge at all. Some variation in knowledge was attributed to time since amputation. Participants that were either born without a limb or lost a limb as a child (*n* = 3) (current ages 64, 49, 67) and prior to the advent of VCA, for example, considered themselves adapted to their circumstances. Their discussions about transplantation suggested that they had some knowledge about hand transplantation but had not taken time to investigate transplant as an option for themselves because they did not see it as relevant to their situation.

Participants suggested that the challenges associated with insurance coverage for treatment or loss of work due to time needed for rehabilitation and recovery was also a concern with any treatment and may contribute to a lack of seeking information about transplant. Communicating with insurance for coverage of prosthetics was identified as an ongoing challenge and worry about insurance coverage for any treatment was discussed. For individuals that see themselves as productive and capable, the loss of work time and rehabilitation burden on family was also identified as a transplant consideration.

“… *it takes me back to when people would come to the bedside to talk to me about, ‘Wanna transplant this, wanna transplant that, and we can take your thumb, and we can take your fingers, and we can take ligaments from the other side,’ you know all these conversations. And anybody else that came into the room was speaking the same language as they were. And I wanted to hear an advocate for the prosthetics, when that was a possible action, and an advocate for all the other options, not somebody who was presenting [all] the options under the umbrella of the one choice*…*” (male, 71, arm shot off in active duty 15 years prior and reattached).*

*“I guess this is all a part of like, making the justification for me to get a better hand or arm, and I guess we just have to keep doing this and going through these processes and adjusting what I have so the insurance will see that we’ve tried because I - I guess there are arms out there now that you can control grasp. I’ve seen it online - stuff where they actually do implants where they can implant sensors into your arm so like if you open a hand or close a hand or move your index finger, you could do all of that with a prosthetic. So, like I guess if it were ever to get that advanced, and my insurance would pay for it*… *I’ve been denied everything, and it was like jumping through hoops just to get me that myoelectric arm that I have*…*Everything’s been just so hard.” (male, 38, bilateral loss to infection 2 years prior).*

### Aging of patient and caregivers and overuse injury worries

When asked about concerns participants had about transplantation, participants organically shared their worries about their age or about aging. They voiced concerns about their own aging and their caregivers aging. With over half the study participants being over the age of 60, many expressed they felt the complexity of the VCA surgery, the recovery time, and the risks associated with lifelong immunosuppression were too much for them at their current age. Three individuals, however, felt they may have considered transplantation if they were younger. Fears of growing older, overuse injuries, and aging primary caregivers are also prevalent.

*“I guess the only thing is the fear of aging. Right now, I’m able to do this stuff, and I have a husband who’s gonna do this stuff. But what happens when he can’t or isn’t around. As I get older, I’m not sure - now I’ve certainly thought a lot about it and looked at a few different things, and my mother is gonna be 90 next year, and she and I have been visiting some senior care facilities, and that’s probably where I’ll end up. But it’s still very unnerving where really, up until this happened, I wasn’t that scared of retiring or even living in a senior facility. But now I’m like well - somebody else is gonna have to dress me and help me and all - do all that stuff.” (female, 64, sepsis, bilateral 4 years prior).*

*“Missing the limb, yep*…*I’m 50 now, and the thing I gotta think about is I don’t wanna wear this one out. My biggest fear is I tear a rotator cuff and then your arm is immobilized for three months, and who knows what the outcome is gonna be. What do I got to use?” (male, 50, accident - lost limb at age 28).*

*“If they actually get to the point where they are doing transplants, if it’s not an issue and insurance covers it, I definitely would get on the list. I’d be interested in doing it. I’d check it out just because thinking down the road, you know, like when I get older and I’m not as active or as strong as I am now, life’s probably gonna get a little harder… That’s the only thing that I really think of is just when I get older and if I’m gonna be able to do the things that I’ve learned or that I’m able to do now.” (male, 38, bilateral lost to infection 2 years prior).*

*“It’s just it eventually comes up–“Okay, I’m getting carpal tunnel.” I go, “Gee, imagine that. Only have one hand and you type all day. What do you think you’re going to get?” Well, part of it being, you have to look at my age. Do I need something that’s that complicated at this stage in my life? So, that’s how I looked at it. Now, had I been 30 years old, that would have been a whole different ball game, you know?” (female, 67, gunshot accident as a child*).

### Perceptions of risks and benefits of vascular composite allotransplantation

Participants described added functional use and dexterity as possible benefits of hand transplantation, but also specified that they would only consider VCA if the function was above and beyond the function gained from their current state or with current prosthetics. Some valued the potential for sensation which can only be gained with VCA, including the greater ability to express emotion and care for others (e.g., hugging their grandchildren). Others described the benefits of social perceptions of having a hand, including looking “normal.” Participants also voiced concern about having the transplant rejected and the potential suffering of having to go through amputation again.

*“And it’s like I can’t imagine being able to do most things for myself to go back to being bedridden and needing all that help from everyone until you can actually use your arms” (male, 38, bilateral lost to infection 2 years prior).*

*“I mean when you’ve been through the rejections stuff and, basically, the therapy and how long it would take to recover, that was probably the biggest thing for me, where they’re talking, and you could be down for a year just trying to let it heal and rehabilitate. I have a young daughter. I’m not going to be out of anything for a year. That can’t happen. I mean I’ve got to be there for her, and that’s the #1 thing I’m going to do, and I can’t walk away from my farm” (male, 49, congenital hand, add’l wrist amputation 2 years prior due to cancer).*

*“Do you want to do this for the rest of your life? And what happens if–I mean I don’t know much about this immunosuppressant-type drugs or anything like that; but if you have to take them for the rest of your life, what happens if you can’t take them for the rest of your life? Does your hand fall off?” (female, 67, gunshot accident as a child).*

## Discussion

In this qualitative study of people living with limb loss, participants had adapted to their loss, finding creative ways to modify their activities of daily living and reducing their reliance on others to assist. They discussed their adaptations using prostheses and their knowledge about different treatment options, including VCA. They had variable degrees of seeking out knowledge about VCA and its requirements. We found that some participants had not explored other treatment options, such as VCA, because of concerns about financial burden or lack of insurance coverage. Our findings are consistent with Talbot et al. (2019) who found that adjustment to amputation (limb loss) was inversely related to an interest in transplantation and concluded this may help explain the difficulty in identifying and selecting candidates for VCA. One possible solution for addressing concerns about financial burden or insurance coverage is to have clearer communication early in the evaluation or treatment process about financial burdens and what insurance will and will not cover. This communication should include all options, including VCA and prosthetics, so fully informed decisions can be made.

Our data indicate that there is worry and concern around aging as well as overuse and injury of existing limbs. Other studies have also shown issues related to overuse injury in the population of people with amputation (Jones and Davidson, 1999; Burger and Vidmar, 2016; Cancio et al., 2021), however, our study adds to this literature by addressing how VCA is perceived in relation to issues of aging and overuse. Future research should explore how incorporating issues of aging and overuse into shared decision making about treatment choices and throughout the healthcare journey affects patient satisfaction and quality of life.

This study identifies the need for ongoing education throughout the healthcare journey of individuals with limb absence. Throughout their lives, as needs change with age, injury, and caregiver access, there needs to be changing and adapting education provided to facilitate decision-making. Clinically, healthcare providers can routinely incorporate information about healthcare options and the self-care needed to maintain their treatment choice. Discussing overuse injuries, aging for themselves, and aging of caregivers should begin early, even if the individual is not actively considering transplantation. Included in education should be information for patients that considers adaptations to their limb absence and how those adaptations may be impacted by aging or overuse. Opportunities may also exist for education and mentoring for individuals eligible for upper extremity transplantation. Peer mentorship of new amputees may help individuals see the potential for having productive and capable lives, regardless of treatment option chosen. These conversations may also include topics such as loss of work time and rehabilitation burden on family that some may or may not consider when discussing treatment options.

With increased attention on the importance of standardized evaluation for amputees and VCA recipients, our results also suggest that routine assessment of quality of life for treatment options may be useful. Ability to adapt and contentment of current treatment option are additional factors that may be especially important in this population and may impact decision-making and help to capture concerns about aging and overuse. Furthermore, to assure that thorough information is provided for current and future decision-making, education about treatment choices should be standardized. Routine standardized psychosocial assessment and education should support current and future decision-making about treatment choices, including VCA, for all individuals with limb loss.

### Limitations

Noted limitations for this study are its small sample size, which is typical of some qualitative interview studies, especially in unique populations such as this convenience sample of individuals that are difficult to find and recruit from the general population Nonetheless, this sample of ten participants represents unique perspectives from a broad range of amputees that can be considered experts on their own healthcare needs and decision making; and the goal of this study was discovery of new information, which can be achieved through small sample size. Future studies that ethnographically capture the changes and trajectory of quality-of-life issues over time or the impact of educational interventions to reduce ambiguity of treatment decision choices would be important next steps for understanding the interconnectedness of treatment choice, selection criteria, assessment of psychosocial health and quality of life and patient functional outcomes.

## Conclusion

Although VCA may be a life-changing option for individuals with loss of an upper extremity, this study highlights individuals who have lived with upper extremity loss for an extended period of time and have adapted their lives around their limb absence as well as individuals who are older and may be less motivated or less interested in limb transplantation. It also indicates the low level of VCA knowledge among these individuals. Lack of awareness and knowledge for this study population indicates an opportunity for education about transplantation earlier. With an increased need to develop evidence-based standardized protocols to evaluate hand transplant candidates, this study suggests the need to include in standardized evaluation: assessment of knowledge base; concerns and insight around aging for amputees; and evaluation of those highly adapted individuals who may be content in their life and may not consider their quality of life improved with a hand transplant. This study identifies the need for ongoing education throughout the healthcare journey of individuals with limb absence. Throughout their lives, as needs change with age, injury, and caregiver access, education should be adapted to support individuals’ decision making around VCA and other treatment options.

## Data availability statement

The datasets presented in this article are not readily available. Due to the nature of this qualitative study, the participants are recruited from a small population sample. De-identified transcripts may contain contextual information on the participant, their lived experience, and their care that could be inadvertently identifiable. To protect these human subjects, these datasets will not be made available. Requests regarding the datasets should be directed to DW, finnie.dawn@mayo.edu.

## Ethics statement

The studies involving human participants were reviewed and approved by Mayo Clinic IRB. The patients/participants provided their written informed consent to participate in this study.

## Author contributions

KS, DF, and JG collected the interview data. JG and DF performed the qualitative analysis. DF wrote the first draft of the manuscript. JG wrote sections of the manuscript. All authors contributed to conception and design of the study and manuscript revision, read, and approved the submitted version.
